# A Quantitative Golgi Study of Dendritic Morphology in the Mice Striatal Medium Spiny Neurons

**DOI:** 10.3389/fnana.2017.00037

**Published:** 2017-04-28

**Authors:** Ivana Bicanic, Ana Hladnik, Zdravko Petanjek

**Affiliations:** ^1^Department of Anatomy and Clinical Anatomy, School of Medicine, University of ZagrebZagreb, Croatia; ^2^Department of Neuroscience, Croatian Institute for Brain Research, School of Medicine, University of ZagrebZagreb, Croatia

**Keywords:** dendritic morphology, basal ganglia, GABA, projection neurons, Huntington’s disease

## Abstract

In this study we have provided a detailed quantitative morphological analysis of medium spiny neurons (MSNs) in the mice dorsal striatum and determined the consistency of values among three groups of animals obtained in different set of experiments. Dendritic trees of 162 Golgi Cox (FD Rapid GolgiStain Kit) impregnated MSNs from 15 adult C57BL/6 mice were 3-dimensionally reconstructed using Neurolucida software, and parameters of dendritic morphology have been compared among experimental groups. The parameters of length and branching pattern did not show statistically significant difference and were highly consistent among groups. The average neuronal soma surface was between 160 μm^2^ and 180 μm^2^, and the cells had 5–6 primary dendrites with close to 40 segments per neuron. Sholl analysis confirmed regular pattern of dendritic branching. The total length of dendrites was around 2100 μm with the average length of individual branching (intermediate) segment around 22 μm and for the terminal segment around 100 μm. Even though each experimental group underwent the same strictly defined protocol in tissue preparation and Golgi staining, we found inconsistency in dendritic volume and soma surface. These changes could be methodologically influenced during the Golgi procedure, although without affecting the dendritic length and tree complexity. Since the neuronal activity affects the dendritic thickness, it could not be excluded that observed volume inconsistency was related with functional states of neurons prior to animal sacrifice. Comprehensive analyses of tree complexity and dendritic length provided here could serve as an additional tool for understanding morphological variability in the most numerous neuronal population of the striatum. As reference values they could provide basic ground for comparisons with the results obtained in studies that use various models of genetically modified mice in explaining different pathological conditions that involve MSNs.

## Introduction

The medium spiny neurons (MSNs) are the most numerous striatal neurons. Data about their morphology are needed to understand basal ganglia circuitry structure and function in normal conditions and in related disorders (Yamada et al., 2016). Changes or loss of MSNs represent a major pathological correlate of several neurological diseases, such as Huntington’s disease, Parkinson’s disease and Lewy Body Dementia (Looi and Walterfang, 2013), for which mice experimental models have been established. Morphological studies using Golgi, as well as intracellular staining, have qualitatively described various MSNs cell types in the striatum of mouse (Rafols et al., 1989), rat (Preston et al., 1980; Kawaguchi et al., 1990), monkey (DiFiglia et al., 1976) and human brain (Braak and Braak, 1982; Graveland et al., 1985). These studies have provided quantitative data about dendritic lengths, branching patterns and dendritic spine densities showing morphological similarities among the molecularly different MSNs subtypes (DiFiglia et al., 1976; Kawaguchi et al., 1990) but without providing detailed data about dendritic morphology, particularly individual segment parameters (Uylings and van Pelt, 2002; van Pelt and Uylings, 2002).

A computer based microscopical system for three-dimensional reconstruction of neurons was developed in order to obtain numerical data of neuron morphology which provided objective information about dynamics of developmental changes or alteration found in different pathological states or genetically modified models (Overdijk et al., 1978; Halavi et al., 2012; van Pelt et al., 2014; Koyama et al., 2015). Up to date the most commonly used is the Neurolucida software (Glaser and Glaser, 1990), which has allowed measuring procedure to become standardized across different laboratories and to create an integrated free-available data base for parameters of neuronal morphology (Ascoli et al., 2007; Peng et al., 2015). Most of the data are obtained from classical Golgi studies. When compared to recently developed intracellular staining (Groc et al., 2002; Cerminara et al., 2013; Unzai et al., 2017), the Golgi staining is more easily adoptable and therefore remains the key method to study neuronal morphology *in vivo* (Petanjek et al., 2008, 2011; Koyama and Tohyama, 2013; Zaqout and Kaindl, 2016).

Over the years the variations in protocols developed in different laboratories have made classical Golgi method inconsistent with lack of uniformity (Braak and Braak, 1985; Bayram-Weston et al., 2016). A potential for eluding this shortcoming has been encouraged by a recently developed commercially available kit-based Golgi–Cox method (FD Rapid GolgiStain Kit; FD NeuroTechnologies, Inc., Ellicott City, MD, USA; Koyama, 2013; Koyama and Tohyama, 2013), although no information proving consistency of the obtained data have been described in the literature so far. In order to establish the consistency of parameters of dendritic morphology by using the above mentioned method, it was necessary to perform an analysis where experimental groups were raised and stained at different times. For this purpose we have made a detailed 3D reconstruction and compared the parameters of dendritic morphology for MSNs in three sets of experiments.

Dendritic length and branching pattern is a ground of microcircuitry organization (Hamilton et al., 2012; Rees et al., 2017), and this research has provided data that could be used as reference values (Brown et al., 2011) for studying MSNs in various models of genetically modified mice.

## Materials and Methods

### Animals, Tissue Preparation and Staining

In this study we have used 15 C57BL/6 wild type mice (7 females and 8 males) kindly provided by the Max Planck Institute for Evolutionary Anthropology in Leipzig, Germany (Enard et al., 2009). Animals were 75–95 days old (13 mice), and 2 animals were older (254 and 305 days). In the Table 1 data on sex and age of each animal used in this study is provided. Animal weight at sacrifice was inside ±8% of established average norm for C57BL/6 wild type mice of corresponding age and sex. Animals were kept together with mothers in the cages for the first 4 weeks of life.

**Table 1:** **Data about sex and age of the animals used in this study**.

Animal number	Sex	Age
1–1	F	254
1–2	M	305
1–3	F	75
1–4	M	77
1–5	M	77
1–6	F	77
**Group 1**	**3M/3F**	
2–1	M	92
2–2	M	89
2–3	F	77
2–4	F	77
**Group 2**	**2M/2F**	
3–1	F	95
3–2	M	95
3–3	M	95
3–4	M	89
3–5	F	83
**Group 3**	**3M/2F**	

*In the table sex (female, male) and age in days for each animal is showed*.

Brain tissue was processed separately in three sets of experiments performed at different times and defined as group 1 (*n* = 6), group 2 (*n* = 4) and group 3 (*n* = 5). All work with the animals used in this study was performed in accordance with the governmental and institutional ethical guidelines and with the approval of Ethics Committee from Max Planck Institute for Evolutionary Anthropology in Leipzig and School of Medicine University of Zagreb.

Animals were deeply anesthetized with sodium pentobarbital injection (60 mg/kg, i.p.) before euthanizing. Brains were not perfused and were removed quickly from the skull to avoid any damage to the tissue. After rinsing, the tissue was sliced in approximately 10 mm thick blocks. The blocks were stained with the FD Rapid GolgiStain™ kit (FD NeuroTechnologies, Ellicott City, MD, USA). They were first immersed in the impregnation solution (A and B) which was replaced after 6–12 h and then kept in dark for 15–16 days. Afterwards, the blocks were put in Solution C which was replaced after 24 h and kept in dark for the next 48–60 h. Cryomicrotome (Microm Thermo Scientific, Walldorf, Germany) was used to cut 200 μm thick slices. Slices were mounted on a gelatin-coated microscope slides, stained, and dehydrated and coverslipped with Permount. The tissue obtained from group 3 was additionally treated with Toluidine blue stain before dehydration.

Tissue preparation and staining were all done by the same person (U.B.) following the FD Rapid GolgiStain™ kit manufacturer’s protocol.

### Dendritic Tree Reconstruction

In total 162 MSNs were three-dimensionally reconstructed using motorized microscope-computer based system and the Neurolucida software version 10 (MBF–Bioscience, Williston, ND, USA). System was composed of *z*-axis motorized Olympus BX61 microscope equipped with *x-y* motorized stage guided by MAC5000 stage controller (Ludl Electronic Products Ltd, Hawthorne, NY, USA).

**Figure 1:**
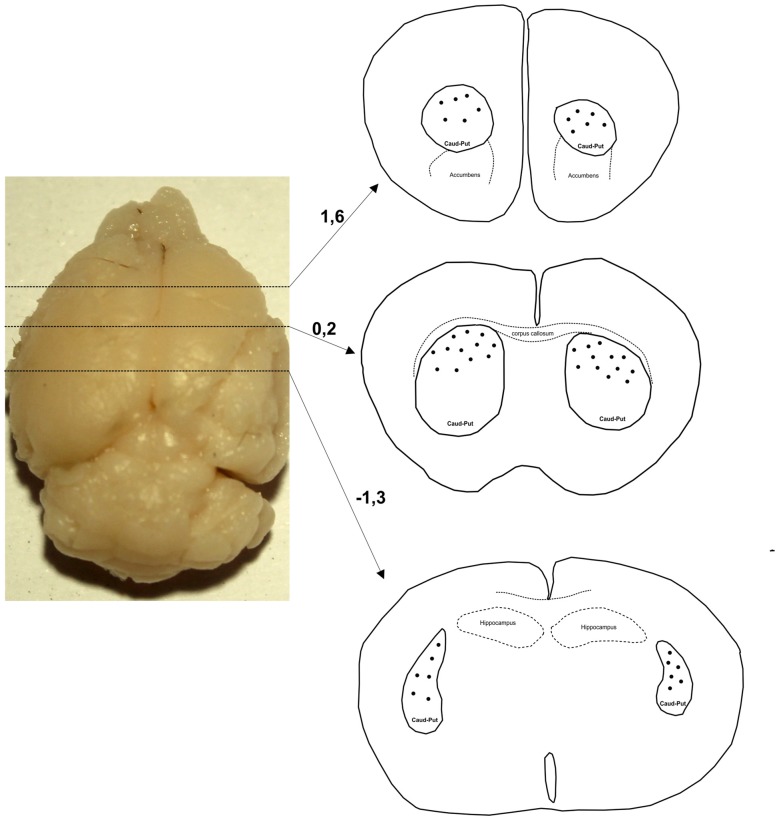
**Levels along the rostro-caudal striatal axis where the neurons were chosen for 3D reconstruction**. Number indicates the position of section in relation to bregma (mm). Distribution of representative neurons is indicated by dots that are inside the caudate-putamen complex (encircled by a full line and indicated as caud-put).

Only neurons with cell bodies located in the dorsal part of striatum within the middle third of the section thickness were included in the analysis. Figure 1 shows levels along the rostro-caudal striatal axis where the neurons were taken for the analysis. In Golgi–Cox slices neurons that lie in the middle third of the section thickness have the highest level of impregnation (Koenderink and Uylings, 1996), and we used this criterion to reduce the number of segments cut at the section surface. The reconstructions were done using a 40× air objective, and neurons were drawn over a live picture on a PC. The cells with somato-dendritic morphology typical for the MSNs and with high density of dendritic spines without any sign of incomplete impregnation (Williams et al., 1978; Braak and Braak, 1985) were reconstructed.

### Morphometry

By using a Neurolucida software extension NeurolucidaExplorer, from each reconstructed neuron the following parameters of somato-dendritic morphology have been extrapolated to numeric values: (1) cell surface; (2) number of primary dendrites; (3) total number of segments; (4) total dendritic length; (5) average length per dendrite; (6) average number of segments per dendrite; (7) average length and volume of individual intermediate (branch); (8) terminal; and (9) incomplete segment.

For the analysis of segment length and volume, each segment was defined as branch (intermediate), terminal, or incomplete (Uylings et al., 1986, 1994). The terminal segments represented segments between the end point of a dendrite and the last bifurcation point before it. The end of a dendrite was defined as regular when it did not finish in the precipitate or when it was not cut at the surface of the tissue slice. Branch (intermediate) segments were segments between the dendritic origin and the first bifurcation point, or between two consecutive bifurcation points. Incomplete segments were segments which could not be traced until the end point of a dendrite because they were cut at the surface of a tissue slice or running into the precipitation.

Scholl analysis was performed to analyze dendritic length and number of intersections per concentric circles starting from the point at the centroid of the cell body. Data were analyzed per each 10 μm and 20 μm concentric circles, excluding the data from the starting circle which was in both cases 10 μm in diameter.

### Statistical Analysis

All data in this study were shown as mean ± standard deviation (SD) per group using average values per neuron as raw data. We applied the STATISTICA software for statistical analysis. The dendritic variables were tested separately using one-way analysis of variance (Conover and Iman, 1981; Uylings et al., 1989). The *a posteriori* Student-Newman-Keuls test for multiple comparisons was applied to determine if the differences among the groups were statistically significant. A *p-level* lower than 0.05 was considered as statistically significant.

## Results

**Figure 2:**
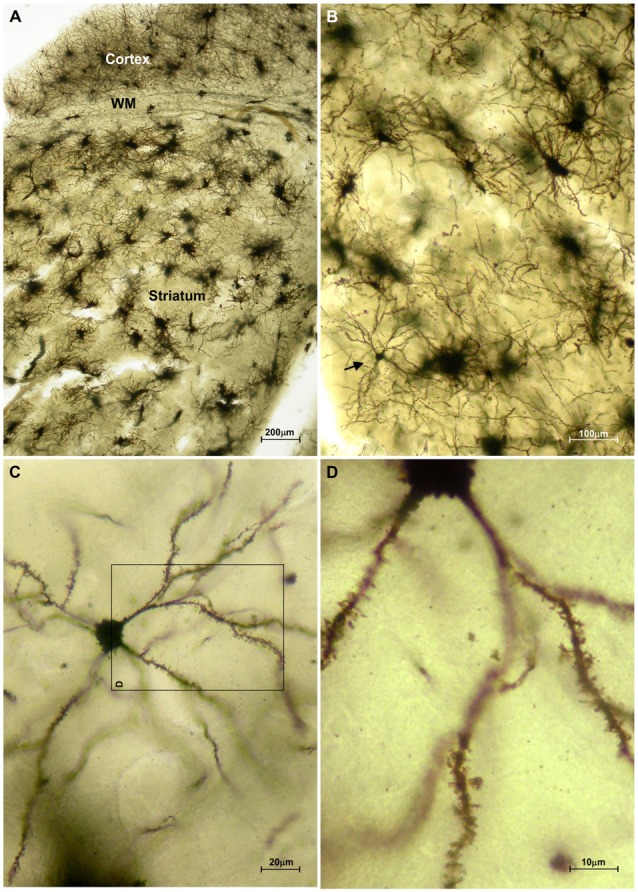
**Representative microphotographs of the mice striatum stained with FD Rapid GolgiStain™ kit. (A)** Overview of striatum at a low power magnification showing domination of regularly distributed tufted cells which staining mainly corresponds to glial cells. White matter fibers that are extension of corpus callosum (CC) are dividing striatum from the cerebral cortex. **(B)** A medium spiny neuron (MSN; arrow) on a higher magnification inside numerous tufted glial cells. **(C)** Typical MSN indicated in **(B)** shown at high magnification with medium sized round soma (around 15 mm in diameter) and spiny dendrites bifurcating in all directions. Panel **(D)** is a magnified square indicated on **(C)** (rotated 90° clockwise).

**Figure 3:**
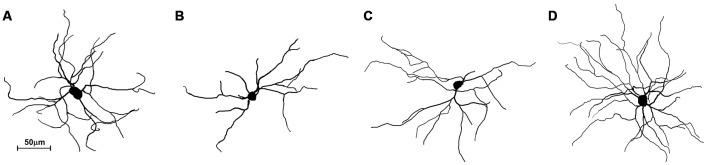
**Three-dimensional reconstructions of the mice MSNs using Neurolucida Software showing different somato-dendritic pattern. (A)** MSN with a relatively large cell body and with an average size and branching pattern of dendritic tree. **(B)** MSN with smaller cell body and only two dendrites having lower branching pattern. **(C)** MSN with smaller cell body and with an average size and branching pattern of dendritic tree. **(D)** MSN with an average cell body and higher number of well bifurcating dendrites. These reconstructions present variations of somato-dendritic pattern which has different prevalence in histological sections.

The striatum was well impregnated, and glial cells could be easily recognized with their typical morphological profile like tufted clumps (Figure 2A). An examination under higher magnification (Figures 2B–D) revealed that the most common neuronal type impregnated in the striatum was MSN. The vast majority of these spiny neurons had medium sized soma and spine laden dendrites, increasing their density gradually towards the more distal segments (Figure 2D). Some of the axons could be followed for a distance that extended well beyond the dendritic field, giving many collateral branches. These cells were considered as MSNs and were taken for quantitative analysis. However, variations in somato-dendritic pattern of MSNs were also observed. Differences among the MSNs were mainly related with the number and complexity of dendrites in relation to the size of their cell bodies (Figure 3).

**Figure 4:**
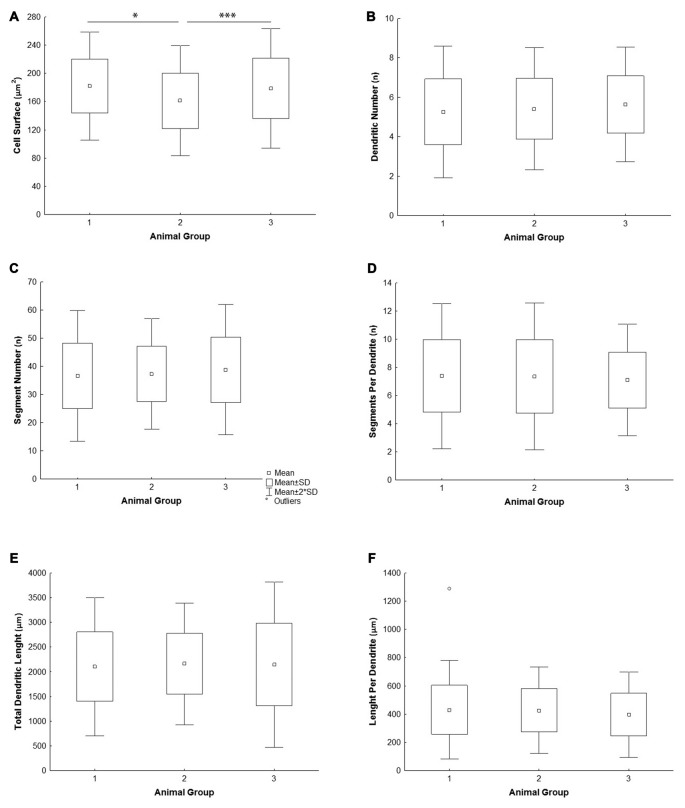
**Graphical representation of the main morphometric values of mice MSNs obtained in three experimental groups**. Quantitative data are presented in columns as mean values, and standard deviation (SD) is indicated by error bars. Measured values are presented as average values per individual neuron **(A–C,E)** or individual dendrite **(D,F)**: **(A)** cell surface, **(B)** total segment number, **(C)** number of primary dendrites, **(D)** number of segments per individual dendrite, **(E)** total dendritic length, **(F)** length per individual dendrite. Statistically significant differences among three groups analyzed are shown by asterisk (*between group 1 and 2; ***between group 2 and 3).

**Figure 5:**
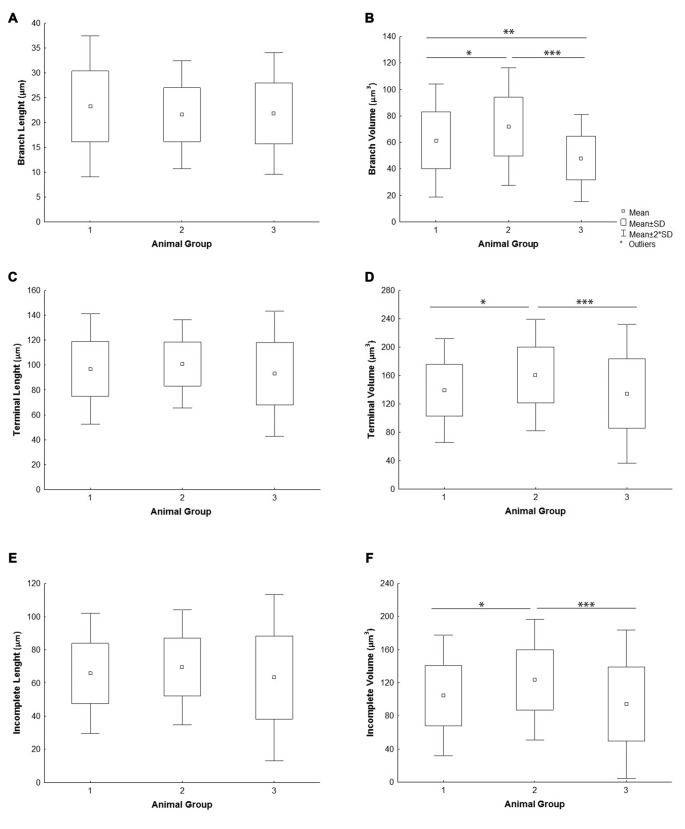
**Graphical representation of individual dendritic segment length and volume of mice MSNs obtained in three experimental groups**. Quantitative data are presented as mean, standard deviation (SD) and statistically significant difference among the three groups analyzed. All measured values are presented as average values per individual segment: **(A)** length and **(B)** volume of intermediate (branch) segment, **(C)** length and **(D)** volume of terminal segment, **(E)** length and **(F)** volume of incomplete segment. Statistically significant differences among three groups analyzed are shown by asterisk (*between group 1 and 2; **between group 1 and 3; ***between group 2 and 3).

All the parameters of somato-dendritic morphology quantitatively analyzed are shown as mean values for each experimental group in Tables 2, 3. Quantitative data of dendritic morphology were in line with the definition of MSNs as medium sized cells with soma surface around 160–180 μm^2^ (Figure 4A). The parameters influenced only by length or branching pattern varied minimally among groups. The cells had in average 5–6 primary dendrites (Figure 4B), close to 40 segments (Figure 4C), and around seven segments per dendrite (Figure 4D). Total dendritic length (Figure 4E) was around 2100 μm, and the length per individual dendrite (Figure 4F) was around 420 μm. The average length of individual segment was around 22 μm for the intermediate segment (Figure 5A), for the terminal segment (Figure 5C) around 100 μm, and for the incomplete segment (Figure 5E) around 65 μm. The values of standard deviation indicated homogeneous distribution of sample.

**Table 2:** **Parameters of dendritic morphology that show consistency among analyzed groups**.

	Mean (SD)		
	Group 1	Group 2	Group 3
1. Dendritic number (*n*)	5.2 (1,6)	5.4 (1,5)	5.6 (1,4)
2. Segment number (*n*)	36.6 (11,6)	37.4 (9,8)	38.8 (11,5)
3. Segments per dendrite (*n*)	7.38 (2,5)	7.36 (2,6)	7.09 (1,9)
4. Total dendritic length (μm)	2014.7 (700,3)	2159.4 (614,8)	2146.4 (836,8)
5. Length per dendrite (μm)	430.6 (173,9)	426.1 (153,0)	396.1 (151,0)
6. Terminal segment length (μm)	96.9 (22,1)	100.9 (17,7)	93.1 (25,1)
7. Branch segment length (μm)	23.2 (7,1)	21.6 (5,4)	21.8 (6,1)
8. Incomplete segment length (μm)	65.7 (18,1)	69.6 (17,3)	63.3 (25,0)

*Values in the table are shown as mean and standard deviation (SD) (brackets)*.

**Table 3:** **Parameters of dendritic morphology that differ among groups**.

	Mean (SD)					
	Group 1	p1–2	Group 2	p2–3	Group 3	p1–3
1. Soma surface (μm^2^)	182.0 (38,3)	**0.02**	161.1 (39,0)	**0.02**	178.8 (42,4)	**n.s**.
2. Terminal segment volume (μm^3^)	139.0 (36,5)	**0.01**	160.7 (39,3)	**0.004**	134.4 (50,0)	**n.s**.
3. Branch segment volume (μm^3^)	61.5 (21,3)	**0.03**	71.8 (22,2)	**0.00001**	48.1 (16,4)	**0.002**
4. Incomplete segment volume (μm^3^)	104.5 (36,4)	**0.01**	123.4 (36,4)	**0.0005**	94.2 (44,8)	**n.s**.

Values in the table are shown as mean and standard deviation (SD). p1–2 indicates p values between group 1 and 2, p2–3 between group 2 and 3 and p1–3 between group 1 and 3. No significant difference was indicated by n.s.

In contrast to the length, the volume of individual segments (Figures 5B,D,F) was significantly higher in group 2, while the soma surface (Figure 4A) was significantly lower. For group 3, in which sections were additionally treated with Toluidin blue stain, the volume of intermediate (branch) segments was significantly lower than in other two groups.

**Figure 6:**
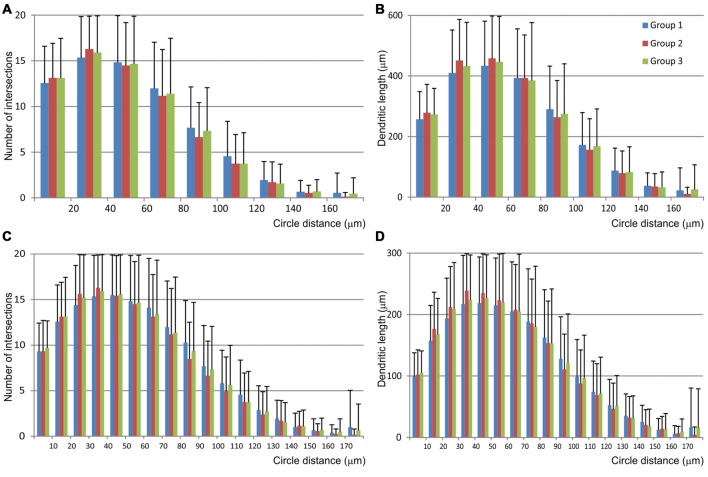
**Graphical representation of data obtained by the Scholl analysis of mice MSNs and compared among three experimental groups**. Number of intersections **(A,C)** and dendritic length **(B,D)** were analyzed per each concentric circle and starting from the point in the centroid of the cell body. Data were analyzed per 20 μm **(A,B)** and 10 μm **(C,D)** concentric circle, excluding the data from the starting circle which was in both cases 10 μm in diameter. Error bar represents standard deviation (SD).

The Sholl analysis (Figure 6) did not reveal any significant differences among groups nor in the length neither in the number of intersections inside particular circle. Most of the dendritic field was located between 20 μm and 70 μm from the soma, and dendrites showed regular branching pattern.

## Discussion

In this study we have quantitatively analyzed dendritic morphology of MSNs in the mice dorsal striatum using Neurolucida system and commercially available FD Rapid GolgiStain Kit and evaluated consistency of numerical values for the obtained parameters.

The data provided here showed that highly consistent parameters of dendritic morphology include the dendritic length and number of segments. The average length of individual segments showed only 5%–8% variation among different experimental groups. However, the parameters of soma surface and volume of individual segments showed statistically significant difference among groups raised and stained at different times. In one of the group the dendritic volume was significantly higher than in the other two groups, at least 15%, while the volume of intermediate segments varied up to 40% between groups. Higher effect on the volume of intermediate than on the volume of terminal segments (Petanjek et al., 2008) could imply different shrinkage of tissue among different sets of experiments, supported further by the fact that the volume was the smallest in the group in which sections were additionally treated with Toluidin blue stain (Koenderink and Uylings, 1996).

We can also not exclude that observed changes in the volume might be related with different functional state of neurons before animal sacrifice. The differences in neuronal activity are first reflected on dendritic thickness, particularly during development. Although the animals were raised by the same protocol, it is impossible to entirely standardize the behavior of mothers and peers in animal groups raised at different times, and thus animals are always exposed to a slightly different social environment. While most of the animals analyzed in this study were sacrificed at an early adult age, the differences among groups obtained from this study might support previous findings that thickness of dendrites is highly influenced by developmental environment (Groc et al., 2002, 2003; Mohammed et al., 2002; Petanjek et al., 2008). Whereas the striatum in adult has been shown to exhibit synaptic plasticity under certain conditions (Fujiyama et al., 2015), our data might also raise the possibility of MSNs being highly responsive to environmental stimulation during development, as previously suggested in rats (Comery et al., 1996).

Presented parameters of cell surface and dendritic length are in line with those obtained in the previous studies performed in mice and rats (Preston et al., 1980; Iwahori and Kiyota, 1987; Rafols et al., 1989; Kawaguchi et al., 1990; Gertler et al., 2008), as well as in other species (DiFiglia et al., 1976; Braak and Braak, 1982; Graveland et al., 1985; Fisher et al., 1986; González et al., 2013). A standard deviation and regular distribution of all parameters supports further that MSNs are homogenous population with quite a uniform morphology and size in all mammals analyzed so far (DiFiglia et al., 1976; Preston et al., 1980; Braak and Braak, 1982; Graveland et al., 1985; Rafols et al., 1989; Kawaguchi et al., 1990), even in reptiles (González et al., 2013).

In this study we have also provided a detailed quantitative analysis of individual segments that has not been provided so far. Length of individual segments is not affected by cutting effect as is the case with total length, and is more stable indicator of changes in dendritic length. The length of intermediate and terminal segments indicates that MSNs have branching pattern similar to the basal dendrites of layer V pyramidal neurons in the human prefrontal cortex (Koenderink and Uylings, 1995), which are less bifurcated than the layer III pyramidal neurons in the same region (Koenderink et al., 1994). When compared to the other Golgi Cox analyses performed in our laboratory, size and complexity of MSN dendritic trees correspond to the size and complexity of basal dendritic trees of the largest pyramidal neurons in the primary motor cortex of mice (Dobrović et al., 2011), but the size and branching pattern of MSN dendritic trees are much larger and more complex than in the pyramidal neurons of the associative cortices and dentate granular cells in rat (Rašin et al., 2011) and mice (Darmopil et al., 2009).

In conclusion, even with the inconsistencies found in dendritic thickness and soma surface that could be methodologically influenced, the parameters of dendritic length and complexity in all three experimental groups were consistent. Hence, the obtained values could serve as reference values for any further research on alterations in MSN dendritic morphology in certain pathological conditions for which various models of genetically modified mice have been used.

## Author Contributions

IB selected histological slices, performed 3-D neuronal reconstructions, organized and statistically analyzed data, wrote a draft and revised the manuscript. AH interpreted data, revised the manuscript. ZP designed the study, prepared histological material, organized microscopical work and prepared quantitative data, guided the statistical analysis, structured and revised the manuscript.

## Funding

This work was supported by the Croatian Science Foundation grant no. 5943 (Microcircuitry of higher cognitive functions, PI: ZP).

## Conflict of Interest Statement

The authors declare that the research was conducted in the absence of any commercial or financial relationships that could be construed as a potential conflict of interest.
